# The Agewell trial: a pilot randomised controlled trial of a behaviour change intervention to promote healthy ageing and reduce risk of dementia in later life

**DOI:** 10.1186/s12888-015-0402-4

**Published:** 2015-02-19

**Authors:** Linda Clare, Sharon M Nelis, Ian R Jones, John V Hindle, Jeanette M Thom, Julie A Nixon, Jennifer Cooney, Carys L Jones, Rhiannon Tudor Edwards, Christopher J Whitaker

**Affiliations:** 1 Research in Ageing and Cognitive Health, School of Psychology, Bangor University, Bangor, Gwynedd LL57 2AS UK; 2 Wales Institute of Social & Economic Research, Data & Methods, Cardiff University, Cardiff, UK; 3 School of Medical Sciences, Bangor University, Bangor, UK; 4 School of Medicine, University of New South Wales, New South Wales, Australia; 5 School of Sports, Health and Exercise Sciences, Bangor University, Bangor, UK; 6 Centre for Health Economics and Medicines Evaluation, IMSCaR, Bangor University, Bangor, UK; 7 North Wales Organisation for Randomised Trials in Health, Bangor University, Bangor, UK

**Keywords:** Goal-setting, Cognition, Cognitive activity, Physical activity, Physical fitness, Diet, Health

## Abstract

**Background:**

Lifestyle factors represent prime targets for behaviour change interventions to promote healthy ageing and reduce dementia risk. We evaluated a goal-setting intervention aimed at promoting increased cognitive and physical activity and improving mental and physical fitness, diet and health.

**Methods:**

This was a pilot randomised controlled trial designed to guide planning for a larger-scale investigation, provide preliminary evidence regarding efficacy, and explore feasibility and acceptability. Primary outcomes were engagement in physical and cognitive activity. Participants aged over 50 living independently in the community were recruited through a community Agewell Centre. Following baseline assessment participants were randomly allocated to one of three conditions: control (IC) had an interview in which information about activities and health was discussed; goal-setting (GS n = 24) had an interview in which they set behaviour change goals relating to physical, cognitive and social activity, health and nutrition; and goal-setting with mentoring (GM, n = 24) had the goal-setting interview followed by bi-monthly telephone mentoring. Participants and researchers were blinded to group assignment. Participants were reassessed after 12 months.

**Results:**

Seventy-five participants were randomised (IC n = 27, GS n = 24, GM n = 24). At 12-month follow-up, the two goal-setting groups, taken together (GS n = 21, GM n = 22), increased their level of physical (effect size 0.37) and cognitive (effect size 0.15) activity relative to controls (IC n = 27). In secondary outcomes, the two goal-setting groups taken together achieved additional benefits compared to control (effect sizes ≥ 0.2) in memory, executive function, cholesterol level, aerobic capacity, flexibility, balance, grip strength, and agility. Adding follow-up mentoring produced further benefits compared to goal-setting alone (effect sizes ≥ 0.2) in physical activity, body composition, global cognition and memory, but not in other domains. Implementation of the recruitment procedure, assessment and intervention was found to be feasible and the approach taken was acceptable to participants, with no adverse effects.

**Conclusions:**

A brief, low-cost goal-setting intervention is feasible and acceptable, and has the potential to achieve increased activity engagement.

**Trial registration:**

Current Controlled Trials ISRCTN30080637

## Background

Lifestyle factors represent prime targets for behaviour change interventions aimed at promoting healthy ageing and reducing dementia risk in the general population. Dementia is one of the major social and economic challenges facing society today [1], with prevalence escalating rapidly as success in tackling and preventing many other health problems leads to greater longevity. There are currently 44.4 million people with some form of dementia worldwide and by 2050 this is expected to increase to approximately 135.5 million [2]. Progress with disease-modifying treatments to date has been limited, and it is increasingly accepted that maximising opportunities for prevention may be a more achievable goal in the medium term. For example, it has been estimated that up to one-third of all cases of Alzheimer’s disease are potentially attributable to seven major modifiable risk factors [3]. Even if the aim were delaying onset, rather than absolute prevention, the benefits would be considerable [4]. Even in later life, a degree of plasticity is retained, meaning that lifestyle and environmental alterations can potentially influence brain health [5].

Progress has been made in the area of secondary prevention, with several risk-reduction programmes focusing on high-risk groups, and either targeting single factors such as physical activity [6] or addressing a range of risk factors [7-9]. However, these approaches typically require costly clinician input and expensive treatment régimes [10], and it may not be feasible to extend them to cover all those who could benefit. As dementia-related pathological processes begin more than a decade before symptoms are first observed, there should be scope for greater benefits if interventions are initiated well before any cognitive difficulties begin to emerge [10]. This suggests that a focus on primary prevention is required in order to establish engaging, low-cost, practical and accessible ways of reducing risk at the population level by promoting healthy ageing [11] along with a specific emphasis on maintenance of cognitive health.

Observational studies and systematic reviews have provided evidence to show that increased engagement in complex cognitive and physical activity, social and cultural participation, and optimisation of cardiovascular health are associated with maintenance of cognitive health and reduced risk of cognitive impairment or dementia [12-15]; however, evidence is limited in some domains, the quality of evidence is generally low, and there is a need for high-quality randomised controlled trials [16]. While most of these factors play a role in prevention of a range of age-related health problems, the role of complex mental activity and cognitive reserve appears more specific to dementia. Engagement in complex mental activity is thought to build cognitive reserve [17], providing a buffer against the effects of dementia-related brain pathology. It has been suggested that increasing cognitive activity and building cognitive reserve would result in significant reductions in incidence of dementia [4,18]. Since many older people are cognitively and physically under-active and socially isolated [19-21], and may have nutritionally inadequate diets [22], all these lifestyle factors represent prime targets for behaviour change interventions that can contribute to strengthening cognitive reserve, stabilising cognitive health, promoting healthy ageing, and reducing risk in the general population.

Trials of physical and cognitive activity have typically involved practice of circumscribed skills for a defined period, producing improvements in trained skills but relatively little evidence of transfer of gains or long-term behaviour change. It may be both more beneficial and more feasible to address multiple factors in an integrated manner [13] with the aim of achieving sustainable changes in behaviour that are integrated directly into everyday life [23,24]. Lifestyle activities can be characterised as the active co-ordination of multiple complex cognitive and physical abilities in order to attain personally-meaningful goals. Increasing activity levels in everyday contexts can ensure that changes are integrated into everyday life and sustained over the longer term, stabilising functioning and improving ability to cope with future challenges to well-being. It is important to develop and test theory-driven interventions aiming to bring about lifestyle changes [12].

### Aims of the study

In this pilot randomised controlled trial we aimed to examine the feasibility and acceptability of a behaviour change intervention based on goal-setting, and to provide preliminary evidence about the efficacy of this intervention in increasing levels of cognitive and physical activity. The theoretical basis for the intervention was in line with recent syntheses of behaviour change models [25], drawing upon social cognitive theories of health behaviour change [26], self-regulation theory [27] and behavioural learning theory [28] as well as incorporating a communication perspective [29,30] and acknowledging the role of non-volitional factors in shaping behaviour. The goal-setting approach was intended to enhance motivation to perform better or maintain effort [31] and was implemented in a context where the environment offered appropriate opportunity and support for lifestyle changes [32-34]. We hypothesised that the goal-setting intervention would lead to increased cognitive and physical activity, with secondary benefits for cognitive, physical, social and psychological functioning, compared to simple discussion of information about activities and health. We also aimed to find out whether providing support and positive feedback by adding ongoing mentoring to the goal-setting would lead to greater improvements than goal-setting alone. The study was intended to provide evidence to support the development of effective ways of promoting health and well-being for older people at the whole population level, which can ultimately contribute to primary prevention of dementia and other forms of age-related cognitive and physical disability.

## Methods

### Design

This pilot randomised controlled trial evaluated the feasibility and acceptability of a goal-setting behaviour change intervention aimed at promoting increased physical and cognitive activity, and provided a preliminary assessment of efficacy and cost-effectiveness (for the trial protocol, see Clare et al. [35]). This was a community-based, person-focused, primary prevention intervention [36], conducted in a rural area of Gwynedd, North Wales, and delivered in the context of a community Agewell Centre for over 50s which offered a range of activities and opportunities for social interaction. All participants provided written informed consent.

Potential participants were invited to join the trial by a member of the research team. They were told that they would have one of two types of interview and that they might or might not receive follow-up phone calls. Those consenting to enter the trial were assessed initially in two sessions. The first, involving questionnaires and neuropsychological tests, covered demographic and background information, physical and cognitive activity, psychosocial well-being, and cognition, and included the measures relating to cost-effectiveness. The second covered physical health, fitness and diet, including anthropomorphic measures, and a blood sample was taken. Once these assessments were completed, participants were randomly allocated to one of the three conditions by the clinical trials unit, using a sequentially-randomised dynamic adaptive computer algorithm which incorporated stratification by gender; married couples were randomised together to the same condition to avoid any cross-contamination. The intervention involved a goal-setting interview, with or without follow-up telephone mentoring; these two conditions (goal-setting, GS, and goal-setting with mentoring, GM) were compared with a control interview involving general discussion about activities and health and information about Centre facilities (information, IC). Following the interview, all participants were free to engage according to personal choice in centre activities throughout the period of their involvement in the trial, as well as accessing any other available community facilities within their local area and undertaking activities of their own choosing. Participants in the GM condition received their allocated mentoring telephone calls. After 12 months, all participants were re-interviewed and follow-up assessments were conducted in two sessions by researchers blind to group allocation. All members of the research team remained blind to group allocation apart from the researcher who conducted the initial and follow-up interviews and the mentoring telephone calls.

The study was approved by the appropriate University ethics committee (Research Ethics Committee, School of Psychology, Bangor University). The trial was registered with Current Controlled Trials, reference ISRCTN30080637. Data collection took place between 1^st^ January 2012 and 30^th^ September 2013. Initial assessments were conducted between 1^st^ January and 30^th^ September 2012, and follow-up assessments between 1^st^ January and 30^th^ September 2013.

### Participants

To be included, participants had to be aged over 50 and living and functioning independently in the local community. All those meeting these criteria who attended the centre were approached as soon as possible after their first recorded attendance and invited to participate in the trial until the target sample size was reached. Reasons for declining to participate were recorded where given. Individuals who were not living and functioning independently (e.g. due to dementia or intellectual disability) were excluded.

### Intervention

On recruitment into the trial, participants were randomly allocated to one of three conditions: information (IC), goal-setting (GS), and goal-setting with mentoring (GM). Each condition involved a one-to-one interview with a researcher lasting up to 90 minutes. IC was a control condition, in which the interviewer discussed information about activities and health and about Centre facilities. In both the GS and GM conditions, participants engaged in a structured goal-setting process to identify up to five goals they wished to work on over the coming year relating to physical activity, cognitive activity, physical health and diet, and social engagement. The goal-setting process was conducted using the Bangor Goal Setting Interview [35]; once goals are identified and clearly expressed in accordance with SMART principles (specific, measureable, achievable, realistic, and timed) [37], current performance, satisfaction with performance, and readiness to change are rated on a 1 – 10 scale where 1 is low and 10 high, and goal attainment indicators representing 25%, 50% and 75% goal attainment are identified. For those in the GM condition, the goal-setting interview was supplemented by five follow-up mentoring telephone calls from the researcher, which took place at bi-monthly intervals; the aim of these calls was to review progress with regard to the selected goals, problem-solve regarding obstacles to progress, encourage and reinforce success, and support maintenance of change. After 12 months, immediately prior to follow-up assessment, all participants were re-interviewed to discuss their experiences over the year, and those in the two goal-setting groups rated current performance with regard to their selected goals, allowing for an evaluation of progress. Following completion of the trial, results were presented at an event to which all participants, centre members and other stakeholders were invited.

### Measures

In order to characterise the sample, demographic information including age, marital status, ethnicity, years and level of education, and socio-economic status, measured objectively in terms of occupation using the standard ONS classification and subjectively using the MacArthur scale of subjective social status [38], was collected from all participants at initial assessment. Indices of social capital (based on ratings of the neighbourhood in which the participant lived), material deprivation (based on possession of common household items) and indices of disadvantage in the domains of civic, social, cultural and leisure participation, social support and contact with others were calculated [39]. The Lifetime of Experiences Questionnaire (LEQ) [40] was used to assess cognitive reserve based on the extent of complex mental activity over the lifespan.

Primary outcomes were physical activity, assessed with the Physical Activities Scale for the Elderly (PASE) [41], and cognitive activity, assessed with the Florida Cognitive Activities Scale (FCAS) [42].

Secondary outcomes covered the domains of psychosocial well-being, cognition, and physical health, fitness and diet. Psychosocial well-being was assessed using the General Self-Efficacy Scale (GSES) [43], Center for Epidemiologic Studies Depression Scale (CES-D) [44], and CASP-19 quality of life measure [45]. Cognition was screened using the Montreal Cognitive Assessment (MoCA) [46], immediate and delayed recall ability was assessed with the California Verbal Learning Test (CVLT) [47], and executive function was assessed with two subtests from the Delis-Kaplan Executive Function System (D-KEFS) [48], Trail-Making and Verbal Fluency. Assessment of physical health, which included anthropometric data, blood pressure and blood sample, provided indices of Body Mass Index (BMI), body fat percentage, and cholesterol level, and yielded the QRISK2 score [49] indicating percentage risk for cardiovascular disease over the next 10 years. Adherence to a Mediterranean diet was assessed with the Mediterranean Diet Adherence Screener (MEDAS) [50]. Physical fitness was assessed with subtests from the Senior Fitness Test (SFT) [51] (up and go; sit to stand) and predicted aerobic capacity was calculated from a submaximal graded exercise step test.

Three questionnaires were included for the evaluation of cost-effectiveness: the EQ-5D [52] measure of health related quality of life, the ICECAP-O measure of capability-related quality of life [53], and an adapted Client Services Receipt Inventory [54], which was used to record participants’ contacts with primary and secondary health and social care services.

A qualitative investigation was also undertaken. Participants’ experiences of and views about the trial were discussed as part of the follow-up interview; this part of the interview, which was audio-recorded and transcribed for further analysis, lasted up to 30 minutes and followed a semi-structured schedule covering the changes experienced by the participants over the year and as a result of taking part, the process and impact of the intervention itself (for those in the goal-setting groups), and views about the conduct of the research. Interviews were conducted between January and September 2013. Seventy people were interviewed; 10 (14%) declined to have the conversation audio-recorded and their responses were noted on the interview schedule for later transcription.

### Planned analyses

Demographic and background information collected at initial assessment was summarised to provide descriptive details of the sample.

For the two goal-setting groups, comparison of goal performance and satisfaction ratings made at initial and follow-up assessments provided an index of progress with achieving goals, and effect sizes (Cohen’s d [55]) were calculated using the difference between the two means divided by the pooled standard deviation; extent of goal attainment was compared using Fisher’s Exact Test. For all three groups, extent of participation in Centre activities was compared using the non-parametric Kruskal-Wallis test.

Primary and secondary outcomes were assessed in two ways. Firstly, for each condition separately, initial and follow-up scores were compared using paired t-tests, and effect sizes (Cohen’s d) were calculated. Secondly, analysis of covariance with baseline scores entered as the covariate was used to assess between-group differences in follow-up scores and calculate effect sizes for two specified contrasts using the contrast estimates divided by the square root of the error mean square term. The first contrast, information (IC) versus the two goal-setting conditions (GS and GM), provided an estimate of the benefits of goal-setting over simply providing information, and the second, GS versus GM, provided an estimate of the extent to which mentoring provided additional benefits compared to goal-setting alone. All variables were examined for homogeneity of variance (Levene’s Test), normality of residuals (Shapiro-Wilk Test) and homogeneity of regression slopes. Three measures violated the homogeneity of variance assumption: GSES; Up and go; total cholesterol. ANCOVA analyses are generally robust to moderate violations of this assumption as long as the sample sizes in each group is approximately equal [56], as was the case for GSES and total cholesterol. Results for the Up and Go test, however, should be interpreted with caution. Five measures violated the normality of residuals assumption: Trail making test T4 – T2; GSES; CES-D; CASP-19; Up and go. However, where differences are not normally distributed but the sample size is greater than 30, the central limit theorem indicates that the usual inference based on the assumption of normality will still be approximately correct. Two measures appeared to violate the homogeneity of regression slopes assumption: predicted aerobic capacity; MEDAS. Results from these tests should therefore be interpreted with caution.

To examine the feasibility and acceptability of the intervention approach, in addition to the information derived from recruitment figures and attrition rates, participants’ views about and experiences of the trial were gathered qualitatively at the follow-up interview, using a structured interview protocol. Interview data were analysed using QSR International NVivo 9 software. Initially two researchers worked independently to generate an initial set of codes from a randomly-selected set of 7 interviews (10% of the total), which included examples from all 3 conditions. The researchers then met to develop a consensus and establish the coding scheme. A further 7 transcripts were randomly selected and independently coded by both researchers. Inter-coder reliability (the number of agreements divided by the sum of the number of agreements and disagreements) was calculated using the ReCal web-based utility [57] as 82%. The remainder of the transcripts was coded by a single researcher. The qualitative data will be reported separately in detail, but key points have been summarised for inclusion here.

A preliminary examination of cost-effectiveness was also undertaken. The cost of the goal-setting intervention was calculated using the number of staff hours spent developing and delivering the intervention. The cost of setting up the centre was annuitized over three years, and the cost of running the centre was calculated from invoices. National unit costs for the price year 2011–2012 were assigned to health and social care services accessed, to calculate a mean total cost per participant [58,59]. Service use costs were not discounted as the follow-up period was one year. The cost-effectiveness of goal-setting (GS and GM combined) compared to control (IC) was evaluated by using EQ-5D values to calculate quality-adjusted life years (QALYs) employing the area under the curve method. A secondary exploratory cost-effectiveness analysis using the ICECAP-O as the measure of effect was conducted. Non-parametric bootstrapping with 1000 replications was used to address the uncertainty associated with point estimates of cost-effectiveness ratios. A summary of the cost-effectiveness findings is included here; these analyses will be reported in more detail in a separate paper.

## Results

**Figure 1:**
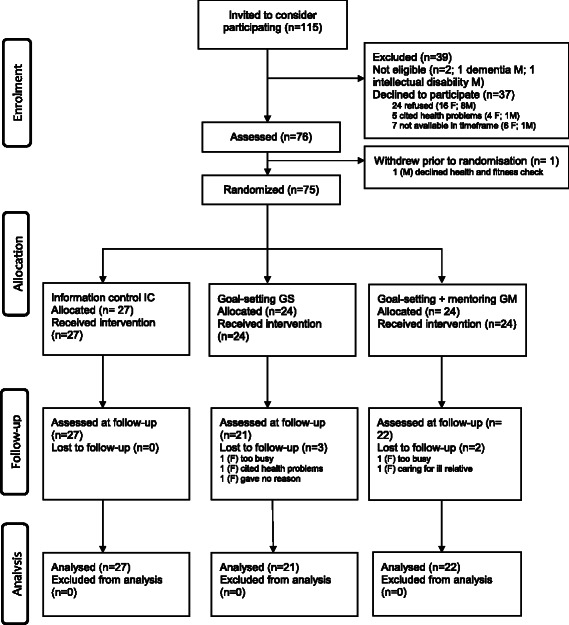
**Agewell trial consort diagram.**

The Agewell trial CONSORT diagram is shown in Figure 1.

### Participants

Characteristics of the whole sample, and of each of the three conditions, are summarised in Table 1. The participants were predominantly female and the sample included 6 married couples. All were either white British or Irish. Approximately one-third lived alone. Over half (58.0%) either had no formal qualifications or had completed secondary school only, and 50.6% had worked in skilled or partly-skilled manual or non-manual occupations. Eighty-eight per cent were owner-occupiers, 96.0% had access to a car, 93.3% owned a mobile phone and 85.3% used the internet; however, two-thirds (66.7%) were classed as having poor social capital, 74.6% as experiencing moderate to high levels of material deprivation, and 42.7% as disadvantaged in relation to contact with others. Nearly one-fifth (18.7%) had caregiving responsibilities. The majority (85.0%) rated their health as good or very good; however, on entry to the study, 43.0% were classed as obese and another 37.0% as overweight, 56.0% were either hypertensive or on medication for hypertension, and 83.0% either had high cholesterol or were on medication for high cholesterol.

**Table 1:** **Sample characteristics for the whole sample and for each group, and between-group statistical comparisons**

	Whole sample	Information (IC)	Goal-setting (GS)	Goal-setting with mentoring (GM)	B	
	N = 75	N = 27	N = 24	N = 24		
	**Mean (SD, range)**	**Mean (SD, range)**	**Mean (SD, range)**	**Mean (SD, range)**	**Kruskal-Wallis**	**P**
Years of education	13.33 (2.93; 9–20)	12.70 (2.91; 9–19)	13.79 (3.18; 9–20)	13.58 (2.68; 10–18)		.
Perceived social status (on 10 rung ladder)	6.53 (1.84; 1–10)	6.51 (1.71; 1–10)	6.54 (1.95; 2–10)	6.54 (1.95; 2–9)		.
	**Mean (SD, range)**	**Mean (SD, range)**	**Mean (SD, range)**	**Mean (SD, range)**		
Age	68.21 (7.92; 51–84)	70.22 (7.77; 52–84)	67.50 (7.66; 52–78)	68.21 (7.92; 51–84)		
LEQ						
Early life	25.08 (6.08; 13–39.8)	23.74 (5.07; 13–31.80)	26.53 (6.63; 15–38)	25.21 (6.08; 13–39.8)	.07	.99
Mid life	28.66 (6.73; 12.5-41.5)	28.49 (6.26; 17.0-39.0)	29.13 (7.23; 16–41.5)	28.40 (6.98; 12.5-27.5)		
Later life	28.11 (4.75; 16.20-28.4)	29.11 (4.23; 21–37.2)	26.97 (4.82; 16.2-33.8)	28.00 (5.20; 18.4-38.4)		
Total	82.08 (14.59; 47.2-108.8)^#^	81.23 (12.94; 51–99.8)	82.77 (16.65; 47.2-108.8)	82.43 (15.04; 60.1-108.8)		
	**N (%)**	**N (%)**	**N (%)**	**N (%)**	**χ** ^**2**^	**p**
Gender						
Male	10 (13.3)	4 (14.8)	1 (4.2)	5 (20.8)		
Female	65 (86.7)	23 (85.2)	23 (95.8)	19 (79.2)		
Marital status						
Single	6 (8.0)	0	2 (12.5)	3 (12.5)		
Married*	39 (52.0)	14 (51.9)	11 (45.8)	14 (58.3)		
Divorced	6 (8.0)	2 (7.4)	3 (12.5)	1 (4.2)		
Widowed	20 (26.7)	10 (37.0)	6 (25.0)	4 (16.7)		
Cohabiting	4 (5.3)	1 (3.7)	1 (4.2)	2 (8.3)		
Living situation						
Living alone	26 (34.7)	12 (44.4)	8 (33.3)	6 (25.0)	2	
Living with others	49 (65.3)	15 (55.6)	16 (66.7)	18 (75.0)		
Socio-economic status						
Unskilled	7 (9.3)	2 (7.4)	4 (16.7)	1 (4.2)	1	
Partly skilled	10 (13.3)	3 (11.1)	4 (16.7)	3 (12.5)		
Skilled manual	7 (9.3)	2 (7.4)	0	5 (20.8)		
Skilled non-manual	21 (28.0)	10 (37.0)	4 (16.7)	7 (29.2)		
Managerial and technical	26 (34.7)	10 (37.0)	10 (41.7)	6 (25.0)		
Professional	4 (5.3)	0	2 (8.3)	2 (8.3)		
Level of Education						
No formal qualifications	23 (30.7)	12 (44.4)	4 (16.7)	7 (29.2)	6	
Secondary school	21 (28.0)	6 (22.2)	7 (29.2)	8 (33.3)		
Vocational training	7 (9.3)	2 (7.4)	3 (12.5)	2 (8.3)		
University degree	15 (20.0)	5 (18.5)	5 (20.8)	5 (20.8)		
Higher degree	9 (12.0)	2 (7.4)	5 (20.8)	2 (8.3)		
Employment						
Retired or unemployed	59 (78.6)	24 (89.9)	19 (79.2)	16 (66.7)		
Employed	16 (21.3)	3 (11.1)	5 (20.8)	8 (33.3)		
Social capital						
Poor	50 (66.7)	19 (70.4)	15 (62.5)	16 (66.7)		
Good	25 (33.3)	8 (29.6)	9 (37.5)	8 (33.3)		
Material deprivation						
None	19 (25.3)	10 (37.0)	7 (29.2)	2 (8.3)	6	
Moderate	40 (53.3)	12 (44.4)	13 (54.2)	15 (62.5)		
High	16 (21.3)	5 (18.5)	4 (16.7)	7 (29.2)		
Disadvantaged						
Civic participation	4 (5.3)	1	2	1	1	.59
Social participation	0	0	0	0		
Leisure participation	6 (8.0)	1	3	2		
Cultural participation	0	0	0	0		
Social support	1 (1.3)	0	1	0		
Contact with others	32 (42.7)	13 (48.1)	11 (45.8)	8 (33.3)		
Subjective health						
Poor/very poor	2 (2.8)	0	0	2 (8.3)	1	
Not too good	4 (5.3)	4 (14.8)	3 (12.5)	0		
Good/very good	64 (85.3)	23 (85.2)	21 (87.5)	22 (91.7)		

*6 married couples participated in the study.

^#^Based on N = 66 (IC – 25; GS – 20; GM – 21) – not all participants were retired at the time of completing the LEQ and hence the post-retirement section could not be completed by these participants.

Recruitment rates suggested that the study was viewed positively, with 66.0% of those approached agreeing to take part. Two men were deemed ineligible to take part as they were not functioning independently in the community: one had dementia and the other had a lifelong intellectual disability. Those approached were not required to provide a reason for declining to take part; however, some cited health problems and others said they were too busy. Five participants were lost to follow-up, an attrition rate of 6.7%. Reasons for withdrawal provided by four of the five were being too busy (2), having health problems (1), and caring for an ill spouse (1). These five participants did not differ significantly in age from those remaining in the study (mean for withdrawals 69.4, mean for completers 68.1, *t* (73) = .34, *p* = .731).

### Changes in goal performance and satisfaction with performance

**Figure 2:**
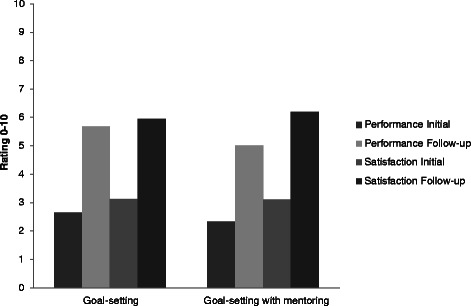
**Ratings (on a 0 – 10 scale) of goal performance, and satisfaction with performance, in the goal-setting and goal-setting with mentoring groups at initial and follow-up assessments.**

**Figure 3:**
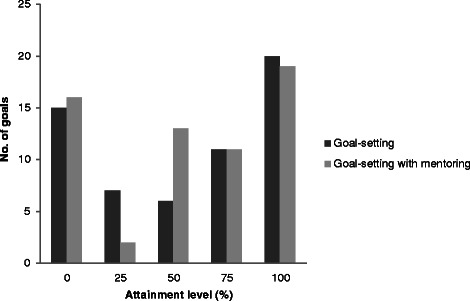
**Goal attainment (%) in the goal-setting and goal-setting with mentoring groups: number of goals meeting each attainment level.**

The participants in the two goal-setting conditions between them set 137 goals (range 1 – 5; mean 2.85 ± 1.2). Goals were classified according to domain; 50 related to physical activity, 40 to physical health and diet, 24 to cognitive activity, and 7 to social engagement, with each of the remaining 16 reflecting a mixture of categories. Examples of goals are shown in Table 2. Ratings indicated that the importance of the four domains to participants’ lives was high (a mean rating of 8.89/10 across all domains) but readiness to change was relatively weak (a mean rating of 3.56/10 across all domains). However, as shown in Figure 2, ratings of performance and satisfaction with performance improved in the goal-setting condition with large effect sizes (performance initial mean 2.65 ± 1.91, follow-up mean 5.69 ± 2.23, effect size 1.5; satisfaction initial mean 3.13 ± 2.02, follow-up mean 5.94 ± 2.07, effect size 1.2), and in the goal-setting with mentoring condition, also with large effect sizes (performance initial mean 2.34 ± 1.61, follow-up mean 5.03 ± 2.34, effect size 1.2; satisfaction initial mean 3.10 ± 1.67, follow-up mean 6.2 ± 2.26, effect size 1.2). The extent to which goals were attained according to behavioural criteria set during the initial goal-setting procedure is summarised in Table 3 and further depicted in Figure 3; this did not differ significantly between the two groups. Overall, 39 goals (28.5%) were fully achieved, and a further 41 (29.9%) met criteria for 50% or 75% attainment.

**Table 2:** **Examples of participants’ goals**

Domain	Goal
Physical activity	I will attend at least a one hour exercise class per week.
	I will be able to run to the end of Nefyn beach.
	I will start swimming and go once a week.
	I will cycle for half an hour each week.
	I will walk one mile on four days a week.
Cognitive activity	I will attend computer classes and learn to email and send attachments.
	In 12 months’ time I will be able to do a moderate Sudoku.
	In 12 months’ time I will be able to save a document on the computer and move it to another file.
	I will do a crossword 3-4 times a week.
	In 12 months’ time I will have completed a 60 credit course.
Diet and health	I will increase my fish intake from once to twice a week.
	I will attend cooking classes at the Centre.
	I will cook my own meals three times a week.
	In 12 months’ time I will have lowered my cholesterol level by 2 points.
	In 12 months’ time I will be a non-smoker.

**Table 3:** **Participation in centre activities and extent of goal attainment**

	Whole sample	Information (IC)	Goal-setting (GS)	Goal-setting with mentoring (GM)	Between-group statistical comparison (Kruskal-Wallis ~ or Fisher’s Exact Test)	
	N = 75	N = 27	N = 24	N = 24		
	**Mean (SD, range)**	**Mean (SD, range)**	**Mean (SD, range)**	**Mean (SD, range)**	**Kruskal-Wallis**	**P**
Centre activities undertaken	2.70 (2.50; 0–13)	2.78 (2.26; 0–10)	2.38 (2.16; 0–8)	2.91 (3.01; 0–13)		.66
Sessions attended	34.00 (35.62; 0–131)	29.07 (35.04; 0–126)	36.34 (38.84; 0–131)	37.50 (37.10; 0–117)		.61
Sessions by type						
Cognitive	11.54 (11.03; 1–43)	6.33 (7.66; 1–30)	14.00 (13.03; 1–43)	14.93 (11.02; 1–35)		.07
Physical	29.60 (26.97; 1–109)	22.00 (20.85; 1–78)	34.85 (29.98; 2–109)	36.72 (31.55; 1–84)		.27
Art and Craft	20.55 (22.32; 1–76)	22.00 (26.61; 1–76)	20.00 (23.35; 3–66)	19.30 (20.08; 1–60)		.92
Number of goals set	2.85 (1.2; 1–5, n = 48)	-	2.83 (1.09; 1–5)	2.88 (1.33; 1–5)		.99
	**N (%)**	**N (%)**	**N (%)**	**N (%)**	**X** ^2^ **(FET)**	**p**
Goal attainment^1^						
<25%	31 (22.6)	-	15 (21.7)	16 (23.5)	5.29	.26
25%	9 (6.6)		7 (10.1)	2 (2.9)		
50%	19 (13.9)		6 (8.7)	13 (19.1)		
75%	22 (16.1)		11 (15.9)	11 (16.2)		
100%	39 (28.5)		20 (29.0)	19 (27.9)		

1. The process of goal-setting included specification of what level of behavioural change would constitute 25%, 50%, 75% and 100% goal attainment. At follow-up, alongside participants’ ratings of performance and satisfaction, the extent of goal attainment was rated for each goal.

2. ~ SPSS v. 20 does not provide values for H but simply reports on whether or not the null hypothesis should be retained, with p values.

### Changes in primary and secondary outcomes

Mean initial and follow-up scores on all measures are summarised in Table 4, which also provides details of within-group comparisons and effect sizes for each condition separately. Table 5 shows the results of the ANCOVA analysis and effect sizes for the two planned contrasts, control (IC) versus goal-setting (GS and GM combined) and GS versus GM.

**Table 4:** **Mean scores on primary and secondary outcome measures at baseline and follow-up in each condition, with effect sizes for benefits at follow-up**

	IC	N = 27					GS	N = 24					GM	N = 24				
Variable	Mean (SD) Range n baseline	Mean (SD) range n follow-up	t	df	p	ES	Mean (SD) range n baseline	Mean (SD) range n follow-up	t	df	p	ES	Mean (SD) range n baseline	Mean (SD) range n follow-up	t	df	p	ES
(max possible score)																		
**Primary outcomes**																		
FCAS (100)	48.25 (8.91)	47.66 (7.95)	.49	26	.62	-.09	45.90 (6.72)	47.14 (8.86)	-.89	20	.38	.19	44.00 (9.22)	45.05 (9.36)	-.76	21	.46	.16
	28.0-60.0, 27	31.0-62.0, 27					24.0-59.0, 24	22.0-63.0, 21					19.0-64.0,24	26.0-61.0, 22				
PASE (361)	117.62 (33.44)	112.96 (46.91)	.48	26	.63	-.09	121.00 (41.85)	124.86 (47.63)	-.43	20	.67	.09	119.17 (50.33)	136.16 (53.47)	−1.57	21	.13	.33
	60.8-214.4, 27	27.1-222.7, 27					38.8-215.0, 24	62.4-226.1, 21					25.0-247.2, 24	69.4-297.2, 22				
**Psychosocial well-being**																		
GSES (40)	31.11 (5.45)	30.96 (6.99)	.14	26	.89	-.02	31.57 (4.96)	31.76 (4.06)	-.17	20	.86	.03	31.45 (3.84)	31.36 (4.38)	.14	21	.89	-.03
	18.0-38.0, 27	16.0-39.0, 27					16.0-38.0, 24	27.0-40.0, 21					22.0-39.0,24	21.0-38.0, 22				
CES-D (60)*	10.62 (7.91)	9.85 (9.43)	.44	26	.66	.08	11.85 (10.45)	10.38 (6.49)	.86	20	.39	.19	7.36 (5.46)	9.73 (6.41)	−1.95	21	.07	-.41
	1.0-34.0, 27	1.0-40.0, 27					0.0-41.0, 24	0.0-23.0, 21					0.0-25.0, 24	0.0-24.0, 22				
CASP-19 (57)	45.51 (6.95)	45.59 (8.27)	-.09	26	.93	.02	42.00 (10.17)	43.90 (9.15)	-.15	20	.16	.32	45.59 (6.61)	45.18 (5.51)	.46	21	.62	.02
	26.0-55.0, 27	22.0-56.0, 27					10.0-57.0, 24	24.0-57.0, 21					26.0-55.0, 24	32.0-54.0, 22				
**Cognitive function**																		
MOCA (30)	25.88 (2.85)	26.37 (2.85)	−1.16	26	.26	.22	25.80 (3.61)	26.23 (3.14)	-.78	20	.44	.17	26.32 (2.64)	27.23 (2.05)	−2.27	21	.03	.48
	20.0-30.0, 27	19.0-30.0, 27					15.0-30.0, 24	16.0-29.0, 21					19.0-30.0, 24	24.0-30.0, 22				
CVLT-II Immediate recall (80)	42.91 (9.17)	43.20 (11.72)	-.17	26	.87	.03	46.10 (9.35)	47.47 (11.08)	-.79	18	.43	.18	48.94 (10.27)	52.35 (11.31)	−1.45	16	.17	.35
	27.0-57.0, 26	22.0-67.0, 24					15.0-64.0, 23	32.0-67.0, 19					27.0-63.0, 24	34.0-72.0, 17				
CVLT-II Delay recall (16)	9.33 (3.21)	10.90 (2.73)	−2.55	20	.02	.56	9.31 (2.23)	10.10 (2.96)	−1.66	18	.11	.38	10.41 (3.00)	11.35 (3.48)	−1.93	16	.07	.47
	1.0-15.0, 25	7.0-16.0, 21					0.0-14.0, 23	6.0-16.0, 19					4.0-15.0, 24	4.0-16.0, 17				
TMT T4-T2 (no max)	69.81 (38.86)	76.44 (42.81)	-.94	26	.35	-.18	69.04 (36.21)	65.47 (31.98)	.49	20	.63	.11	59.18 (30.20)	62.14 (31.32)	-.38	21	.71	-.08
	27.0-57.0, 25	16.0-169.0, 27					19.0-180.0, 24	12.0-129.0, 21					18.0-137.0, 24	20.0-121.0, 22				
VF (no max)	38.59 (10.87)	41.74 (10.74)	−2.05	26	.05	.39	36.96 (14.58)	43.47 (15.24)	−4.15	20	<.001	.90	36.64 (13.92)	39.41 (13.74)	−1.80	21	.09	.38
	15.0-60.0, 27	16.0-61.0, 27					8.0-56.0, 24	10.0-68.0, 21					7.0-60.0, 24	10.0-63.0, 22				
**Physical health and diet**																		
Body fat percentage*	38.57 (9.43)	36.94 (7.53)	1.65	21	.11	.35	38.43 (7.26)	37.08 (6.71)	1.49	18	.15	.34	39.29 (9.94)	37.32 (7.28)	1.44	15	.17	.36
	23.2-69.0, 26	23.0-50.6, 23					22.0-53.0, 23	23.3-47.1, 19					16.0-61.0, 21	23.6-48.4, 17				
BMI*	29.22 (5.49)	28.75 (5.23)	2.17	22	.04	.80	27.92 (4.26)	27.96 (4.41)	-.20	19	.84	.04	29.74 (4.49)	29.00 (4.37)	1.73	18	.10	.40
	21.2-41.8, 27	20.3-40.0, 23					17.7-35.0, 24	18.0-35.8, 20					21.7-39.5, 24	21.2-37.5, 19				
MEDAS (14)	6.51 (2.42)	7.22 (2.13)	−1.96	26	.06	.39	6.33 (2.26)	6.52 (2.46)	-.61	20	.55	.13	6.55 (2.18)	7.18 (1.71)	−1.33	21	.20	.28
	1.0-10.0, 27	1.0-11.0, 27					1.0-10.0, 24	2.0-11.0, 21					1.0-10.0, 24	5.0-11.0, 22				
Total cholesterol*	5.76 (1.20)	5.65 (1.07)	.93	18	.36	.20	6.00 (1.07)	5.57 (1.03)	1.71	16	.10	.42	5.59 (1.07)	5.31 (1.24)	.83	13	.42	.22
	3.4-8.0, 22	3.5-7.7, 22					4.8-8.6, 21	3.8-7.3, 18					3.6-7.8, 20	2.7-6.6, 15				
QRISK*	21.51 (10.33)	20.20 (9.50)	3.39	25	.002	.66	18.02 (10.13)	16.88 (9.92)	2.76	20	.01	.57	17.86 (8.29)	16.46 (8.70)	2.73	20	.01	.60
	4.9-42.5, 27	4.2-36.4, 26					3.3-45.3, 24	5.9-42.3, 21					4.6-34.1, 24	3.6-31.5, 21				
**Physical fitness**																		
Predicted aerobic capacity	18.51 (3.23)	20.09 (4.83)	−1.87	9	.09	.59	18.29 (4.00)	18.29 (3.94)	.001	10	.99	0	17.99 (3.43)	19.55 (5.40)	−2.30	10	.05	.69
	10.6-23.0, 16	14.7-29.2, 10					12.7-18.0, 17	13.6-25.2, 11					12.5-19.1, 13	12.9-30.3,12				
Up and go*	5.38 (.80)	6.67 (3.74)	−1.72	21	.09	-.37	5.03 (.67)	5.05 (1.16)	-.14	18	.89	-.02	5.62 (1.81)	5.37 (1.18)	1.04	17	.31	.25
	3.8-9.1, 26	4.1-18.6, 23					3.8-6.8, 23	3.8-9.2, 19					3.8-12.1, 24	3.94-8.78, 18				
Sit to stand	14.42 (2.39)	14.00 (3.47)	.86	21	.39	-.18	14.89 (2.80)	15.57 (3.62)	−1.34	18	.19	.31	13.94 (2.14)	14.94 (2.43)	−2.66	15	.02	.66
	8.0-19.0, 26	7.0-21.0, 21					10.0-21.0, 23	11.0-25.0, 19					6.0-17.0, 23	10.21.0, 17				

*Lower scores better.

**Table 5:** **Results of ANCOVA and effect sizes for planned contrasts**

Measure	Group	n	Estimated marginal means at follow-up (and standard errors)	Covariate		Significance		Effect size	
(maximum possible score)				F	P	F	p	Contrast 1#	Contrast 2+
								GS + GM vs. IC	GM vs. GS
**Primary outcomes**									
Florida cognitive activities scale (100)	IC	27	46.13 (1.17)	75.75	<.001	.25	.78	.15	-.11
	GS	21	47.38 (1.31)						
	GM	22	46.71 (1.30)						
Physical activity scale elderly (361)	IC	27	113.78 (8.50)	17.78	<.001	1.55	.22	.37	.28
	GS	21	123.85 (9.63)						
	GM	22	136.14 (9.41)						
**Psychosocial well-being**									
General self-efficacy scale (40)	IC	27	31.12 (.87)	33.58	<.001	.07	.93	.07	-.07
	GS	21	31.62 (.98)						
	GM	22	31.3 (.96)						
CES-Depression Score (60)*	IC	27	9.53 (1.29)	23.82	<.001	.37	.68	-.11	-.24
	GS	21	9.45 (1.47)						
	GM	22	11.02 (1.45)						
CASP-19 (57)	IC	27	44.77 (.90)	125.08	<.001	.63	.54	.07	-.34
	GS	21	45.88 (1.03)						
	GM	22	44.30 (.99)						
**Cognitive function**									
MOCA (30)	IC	27	26.44 (.37)	72.38	<.001	.79	.46	.13	.35
	GS	21	26.36 (.42)						
	GM	22	27.02 (.41)						
CVLT-II Immediate recall total (80)	IC	24	45.41 (1.76)	57.02	<.001	1.18	.31	.35	.30
	GS	19	47.09 (1.94)						
	GM	17	49.67 (2.09)						
CVLT-II Delayed recall total (16)	IC	21	11.13 (.49)	47.68	<.001	.61	55	-.25	.20
	GS	19	10.35 (.52)						
	GM	17	10.80 (.55)						
Trail making test T4-T2 (no max.)*	IC	27	74.57 (6.08)	24.63	<.001	.79	.46	.31	.18
	GS	21	64.01 (6.89)						
	GM	22	65.84 (6.76)						
Verbal fluency (no max.)	IC	27	40.81 (1.41)	149.83	<.001	1.65	.19	.17	-.52
	GS	21	43.93 (1.60)						
	GM	22	40.12 (1.57)						
**Physical health and diet**									
Body fat percentage*	IC	22	37.05 (.81)	142.78	<.001	.04	.96	-.01	.09
	GS	19	37.29 (.87)						
	GM	17	36.93 (.95)						
BMI*	IC	23	28.51 (.27)	718.12	<.001	1.31	.27	-.08	.52
	GS	20	28.98 (.29)						
	GM	19	28.75 (.30)						
MEDAS	IC	27	7.19 (.32)	47.19	<.001	.87	.42	-.20	-.32
	GS	21	6.61 (.36)						
	GM	22	7.14 (.35)						
Cholesterol*	IC	19	5.68 (.20)	34.32	<.001	.44	.64	.27	.00
	GS	18	5.44 (.21)						
	GM	15	5.45 (.23)						
QRISK*	IC	26	18.12 (.40)	1388.21	<.001	.13	.88	.07	.13
	GS	21	18.10 (.44)						
	GM	21	17.84 (.44)						
**Physical fitness**									
Predicted aerobic capacity	IC	10	19.79 (.74)	93.31	<.001	1.66	.21	-.31	-.69
	GS	11	18.25 (.70)						
	GM	12	19.87 (.70)						
Up and go*	IC	22	6.64 (.49)	11.93	.001	2.67	.08	.62	.09
	GS	19	5.33 (.53)						
	GM	18	5.13 (.54)						
Sit to stand	IC	21	14.02 (.45)	79.97	<.001	2.50	.09	.61	.16
	GS	19	15.12 (.48)						
	GM	17	15.46 (.52)						

*Lower scores better.

^#^Contrast 1 examines whether goal-setting (either alone or with added mentoring) produced greater benefits than control; a positive effect size favours goal-setting and a negative effect size favours control.

^+^Contrast 2 examines whether adding mentoring to goal-setting produced greater benefits than goal-setting alone; a positive effect size favours goal-setting with mentoring and a negative effect size favours goal-setting alone.

### Cognitive and physical activity

Both the goal-setting and goal-setting with mentoring conditions increased their engagement in cognitive and physical activity, while the control condition decreased very slightly in activity levels. Within-group pre/post comparisons showed a small-to-medium effect size for physical activity in the goal-setting with mentoring condition and a small effect size in the goal-setting condition, and small effect sizes for cognitive activity in both conditions. With regard to the planned contrasts, the effect size for goal-setting compared to control was small for cognitive activity and small-to medium for physical activity. For physical activity, mentoring added further benefits to goal-setting alone, with a small effect size, but for cognitive activity, a small effect size favoured goal-setting alone over mentoring.

### Psychological well-being

Changes in self-efficacy were negligible. Depression mean scores reduced in the control and goal-setting conditions, but increased in the goal-setting with mentoring condition. Small effect sizes favoured control over goal-setting and goal-setting over mentoring. The number scoring at or above the cut-off for clinical depression reduced in the control and goal-setting conditions at follow-up (from 7 to 5 and 6 to 4 respectively) but increased in the goal-setting with mentoring condition (from 3 to 5). Quality of life ratings on the CASP-19 increased in the goal-setting condition, but changes were negligible in the other two conditions; in the planned contrasts, goal-setting was superior to both control, with a very small effect size, and goal-setting with mentoring, with a medium effect size.

### Cognition

All three conditions improved in general cognitive ability assessed with the MOCA screening instrument; the greatest improvement was seen in the goal-setting with mentoring condition. In the planned contrasts there was a small effect size favouring goal-setting compared to control, and a small-to-medium effect size favouring goal-setting with mentoring compared to goal-setting alone. All three conditions improved on immediate recall, with the greatest improvement in the goal-setting with mentoring condition. In planned contrasts, goal-setting led to greater improvement than control, with a small-to-medium effect size, and mentoring added further benefits, again with a small-to-medium effect size. Improvements were also seen in delayed recall; in the planned contrasts, control was superior to goal-setting, with a small effect size, and mentoring was superior to goal-setting, with a small effect size. Two aspects of executive function were assessed. On the trail-making task, only the goal-setting condition improved; the mentoring condition declined slightly and the control condition more markedly. Goal-setting produced improvements compared to control, with a small-to-medium effect size, and goal-setting alone was superior to mentoring, with a small effect size. On the verbal fluency task, all three conditions improved. In planned contrasts, goal-setting produced greater improvements compared to control, with a small effect size, and goal-setting alone was superior to mentoring, with a medium effect size.

### Physical health and diet

All three conditions reduced body fat percentage; hence in the planned contrasts effect sizes were negligible. BMI reduced in both control and mentoring conditions, but increased slightly in the goal-setting condition. Thus there were negligible differences between goal-setting and control, but mentoring added considerable benefits over goal-setting alone, with a medium effect size. QRISK2 score decreased in all three groups; therefore in the planned contrasts there were only very small effects for goal-setting compared to control and for added benefits of mentoring. Cholesterol levels decreased in all three groups. Improvement was greater in the goal-setting conditions compared to control, with a small effect size, but mentoring did not provide added benefits. All three conditions increased adherence to the Mediterranean diet; in the planned contrasts there was a small effect size favouring control over goal-setting, but mentoring produced better improvement than goal-setting alone, with a small-to-medium effect size.

### Physical fitness

Predicted aerobic capacity increased in the control and mentoring conditions, but did not change in the goal-setting condition; in planned contrasts, control was superior to goal-setting, with a small effect size, but mentoring provided added benefits over goal-setting alone, with a medium effect size. Scores on the ‘up and go’ test improved in the mentoring condition, did not change in the goal-setting condition, and declined in the control condition; in the planned contrasts, goal-setting was superior to control, with a medium effect size, and mentoring added limited benefits. Scores on the ‘sit to stand’ test improved in both goal-setting conditions but declined slightly in the control condition; in the planned contrasts, goal-setting was superior to control with a medium effect size, and mentoring provided some added benefits with a small effect size.

### Participation in the centre

Participants’ activity-related goals could be addressed in any setting and were not restricted to centre-based activities. However, participation in centre activities provides one indication of whether activity levels differed between the groups. Details of activity participation are summarised in Table 3. Of the 75 participants, 69 chose to attend the Centre during the year and participate in centre activities. Two men finished their computer courses just prior to randomisation, and did not engage in further activities. Two women did not attend further after randomisation because of childcare commitments, one found it too far to travel, and a fourth took up employment. The mean number of activities undertaken during the year was 3 (range 0 – 13) and the mean number of sessions attended was 34 (range 0 – 131). The GS and GM groups attended more sessions than the IC group, and participated more extensively in cognitive and physical activities; this difference was significant for cognitive activity participation.

### Participants’ experience of the trial

Participants’ accounts suggested that participation in the study, especially for those in the goal-setting conditions, raised awareness and stimulated change; 37 (86%) of those in the two goal-setting conditions, and 17 (63%) of controls, said that participating in the research had raised their awareness of the importance of a healthy lifestyle, and identified specific changes they had made. These changes in activities and lifestyle were said to lead to improved well-being, greater confidence, a sense of purpose, and a feeling of belonging. In some cases, health problems were identified and managed, or existing problems were managed better. Thirty-six per cent of those reporting changes said that spouses, families or friends also benefitted.

Less positive comments related mainly to the assessments. Some participants said the physical fitness and memory assessments were difficult or made them feel anxious, and a few found the health assessment intrusive. In addition, a few people found the goal-setting process challenging. Perceived barriers to making lifestyle changes included illness and hospitalisation, mobility problems, arthritis, joint or back problems, lack of time, lack of access to transport, assuming caregiver responsibilities, and bad weather.

Participants did sometimes talk with others about the research, but there was no evidence that participants identified the different interview types or distinguished the nature of the other conditions. When debriefed about the nature of the three conditions, 10 (37%) of the control participants said that if given the choice they would opt for one of the goal-setting conditions, but overall most participants were satisfied with their allocation.

### Cost-effectiveness

The costs of setting up and running the Centre, and of developing and delivering the intervention, are shown in Table 6. Centre set-up and intervention development costs were annuitized over 3 years, based on up to 600 people using the facilities during this period; records showed that 400 people attended during the first two years of operation. Annual running costs for the centre were calculated. We calculated the total cost per participant of setting up and running the centre and receiving the intervention to be £241.77 for controls, £251.93 for those in the goal-setting condition and £268.86 for those in the mentoring condition. Service use data was available for all 70 participants who completed the 12 month follow-up. The mean costs (including participants’ intervention, health and social care costs), mean QALYs accrued and mean ICECAP-O score changes between baseline and follow-up are shown in Table 7. When setting the cost-effectiveness acceptability threshold at £20,000 per QALY, there was a 65% probability that receiving a goal-setting interview was more cost-effective than receiving an information only interview. There was only a 36% probability that goal-setting with mentoring was more cost-effective than goal-setting alone.

**Table 6:** **Costs of setting up and running the centre, and of developing and delivering the intervention**

Cost of setting up the centre (annuitized over 3 years)			
Task			Cost
ACGM development officer, 50% FTE			£5,377.57
Volunteer training			£356.93
Administration costs			£356.93
Promoting the centre			£356.93
Equipping the centre			£3,390.87
Recruiting centre staff/facilitators			£1,225.50
*Total centre set-up cost*			*£11,600.14*
*Centre set-up cost per person (based on up to 600 attending the centre over 3 years)*			*£19.33*
**Cost of running the centre (per year)**			
Task			Cost
ACGM centre co-ordinator’s salary			£9,000.00
Rent of an office for the co-ordinator			£5,000.00
Management of centre staff			£14,040.94
Rent of the centre			£4,382.50
Administration costs including utility bills and travel reimbursement			£5,699.00
*Annual centre running cost*			*£38,122.44*
*Annual running cost per person (based on up to 200 attending the centre per year)*			*£190.61*
**Cost of developing the goal-setting and mentoring intervention (annuitized over 3 years)**			
Intervention development time			£590.90
Training staff to deliver the interviews and mentoring			£144.80
Supervision of staff delivering the interviews and mentoring			£126.92
*Total intervention set-up cost*			*£862.62*
*Intervention set-up cost per participant (n = 75)*			£11.50*
**Cost of delivering interviews and mentoring**			
	IC	GS	GM
Initial interview cost per person	£20.32	£30.48	£30.48
On-going telephone mentoring cost per person	N/A	N/A	£16.93
*Interview and mentoring cost per person*	*£20.32*	*£30.48*	*£47.41*
**Total cost per trial participant, including centre set-up and running costs and intervention costs**			
	IC	GS	GSM
	£241.77	£251.93	£268.86

Note: IC = control, GS: goal-setting, GM: goal-setting with mentoring.

*For future studies the cost of intervention development time would not apply and hence the cost per person would reflect only the cost of staff training and supervision; this is calculated as £3.63 per participant.

**Table 7:** **Mean costs, mean QALYs accrued and mean ICECAP-O change at 12 months**

	IC (n = 27)	GS (n = 21)	GM (n = 22)
Mean cost (s.d.)	£1,482.00 (£3,495.88)	£1,510.59 (£3,825.71)	£1,432.75 (£2,312.49)
Mean QALYs (s.d.)	0.85 (0.18)	0.87 (0.17)	0.83 (0.24)
Mean ICECAP-O change (s.d.)	−0.01 (0.13)	0.03 (0.07)	0.00 (0.10)

Note: IC = control, GS: goal-setting, GM: goal-setting with mentoring.

## Discussion

In this pilot trial we evaluated the feasibility and acceptability of a goal-setting intervention delivered in the context of an Agewell centre for over 50s, and gathered preliminary evidence regarding efficacy of the goal-setting intervention in promoting increased mental and physical activity and improving well-being, mental and physical fitness, diet and health, and regarding cost-effectiveness. In contrast to studies that tackle individual risk factors in isolation with highly prescriptive interventions, in this trial we opted for an ecologically valid approach, aiming to bring about sustainable behaviour change within a real-world, everyday context tailored to the needs and circumstances of older people in a rural community. The choice of activity, and the amount of time required to be devoted to the activity, were not specified by the researchers [60]; instead, participants in this study made their own choices about activity participation. This was felt to be more realistic in terms of developing an approach that could potentially be scaled up to a wider, population level. Here we first discuss efficacy, followed by feasibility and acceptability.

### Preliminary evidence regarding efficacy and cost-effectiveness

Efficacy was explored in terms of effect sizes, with the aim of gathering data that would inform sample size calculations for future, larger-scale trials. As these effect sizes are based on a relatively small sample, they must be interpreted cautiously [61]. However, they do suggest that the goal-setting intervention produced worthwhile benefits. The two goal-setting conditions taken together increased their levels of physical (effect size 0.37) and cognitive (effect size 0.15) activity, and achieved additional benefits in secondary outcomes over and above those seen in the control condition, showing greater improvements (effect sizes ≥ 0.2) in memory and executive function, greater reductions in cholesterol level, greater improvements in balance and grip strength, and improvements in agility, physical flexibility and aerobic capacity. There was, however, limited impact on measures of psychosocial well-being. Changes in activity level could account for the benefits in secondary outcomes observed in the goal-setting conditions [62]. The cost-effectiveness analysis presented a high probability that goal-setting is cost-effective compared to simple provision of information.

Including the goal-setting with mentoring condition allowed us to examine whether enhancing the goal-setting process with follow-up mentoring telephone calls led to greater benefits than the goal-setting interview alone. Adding follow-up mentoring to the goal-setting produced some further benefits over and above goal-setting alone (effect sizes ≥ 0.2) in terms of greater increases in physical activity levels, greater improvements in memory and body composition, and improvements in global cognition. However, while added mentoring brought benefits in some areas, the goal-setting with mentoring condition fared worse than the goal-setting condition on measures of quality of life and depression (effect sizes ≥ −0.2). This is difficult to interpret in the context of a small sample size, but on balance the evidence from this pilot trial does not offer strong support for adding a mentoring component, and the cost-effectiveness analysis also offered limited support for the benefits of mentoring.

The control condition did not increase activity levels, but showed improvements (effect sizes ≥ 0.2) at 12 month follow-up in global cognition, memory, executive function, diet, body composition, cholesterol level, risk of cardiovascular disease, aerobic capacity and physical flexibility, albeit often to a lesser extent than the goal-setting conditions. Since the controls showed no change in self-reported levels of physical and cognitive activity but did improve to some extent on some secondary outcome measures, increases in activity level cannot account for the other benefits identified in the control condition. This suggests that participation in the Centre in itself was beneficial, and that this kind of community-based resource offers valuable potential for promoting protective behaviours and reducing risk. Overall, while all three conditions benefitted to some degree and in some areas, a specific focus on identifying individual behaviour change goals was required in order to achieve increased activity engagement and to bring about more substantial improvements in cognition, health, diet and physical fitness.

Physical activity serves as a protective factor in relation to a range of age-related conditions. The physical activity domain was a popular choice for goal selection, and in general participants could readily understand the potential value of increasing physical activity. The effect size obtained for self-reported physical activity was greater than the mean effect size identified in systematic reviews of the effects of specific physical activity interventions, whether delivered alone or in combination with other intervention components: 0.19 for healthy adults of any age [63]; 0.26 for duration, frequency or intensity of activity in older people [64]; 0.19 for self-reported increases at 12 months when measured as a continuous scale in people aged 55 – 70 years, or an odds ratio of 1.63 when measured as a dichotomous variable [21]. Interventions that, like ours, focused solely on behavioural strategies such as goal-setting and cueing were more effective than interventions incorporating cognitive strategies such as health education and provision of information [63], and for older people, centre-based programmes had larger effect sizes than home-based programmes [64]. Tailoring interventions to participants via personalised goals or offering environmental support via provision of relevant local information about exercise opportunities is important [21].

Engagement in cognitive activity is thought to be protective specifically in relation to age-related cognitive disability and dementia. This area attracted fewer goals, and tended to be less well represented in the centre activities programme, although computer classes and local history sessions were offered regularly. There may be a need to increase awareness of the value of cognitive activity and its potential preventive benefits; while fear of developing dementia is common [65], awareness of risk and protective factors is limited [66,67]. It is also possible that the measure used to capture cognitive activity engagement was not sufficiently sensitive to change, especially as change might arise in terms of the quality and depth of engagement rather than the number of activities or the frequency with which they are undertaken. We were unable to find comparable intervention studies using engagement in cognitive activity as an outcome measure. Our secondary outcomes yielded an effect size for immediate recall greater than that obtained in a meta-analysis comparing focused memory training interventions to active control conditions (.35 vs. .18) [68], and a similar lack of benefits in delayed recall. Well-designed, focused cognitive training interventions can produce gains specific to the functions trained [69] and follow-up data suggest long-term benefits in maintenance of everyday functioning [70]. However, our expectation was that a focus on lifestyle change involving increased engagement in complex cognitive activity might result in more generalised benefits across domains of cognitive function [60,71,72]; in support of this, our goal-setting conditions also showed benefits in performance on executive function tests.

One key limitation is that evaluation of activity levels was based on self-report questionnaires. In future work, outcome evaluation could be enhanced by the inclusion of more objective data, for example monitoring devices could be used to provide information about activity levels over a period of time. However, secondary outcome measures provided objective assessments of cognitive function and physical fitness, reflecting the underpinning abilities supporting cognitive and physical activity engagement. Differences in outcomes between the goal setting and goal setting with mentoring groups meant that the planned comparison between the control condition and these two groups taken together may not have been the optimal approach to data analysis, and separate comparisons may have yielded useful information. The benefits seen in the control condition suggest that participating in the Centre was beneficial in itself, but the control participants had elected to join the research, and hence might be expected to be among those more likely to benefit from the Centre. Additionally, the assessment process in itself may have increased awareness of physical and mental fitness and health, and of the possible value of lifestyle changes. However, in the two goal-setting conditions the ratings of readiness to change, made soon after the initial assessment, were quite low, even though the specified domains were seen as important; this would tend to suggest that being assessed in itself did not have a big impact on motivation to change. Although at follow-up participants would have been more familiar with what the assessments involved, practice effects on the objective tests included with the assessment were considered unlikely after a 12-month interval. Of those who declined to participate in the trial, some cited health problems, and it may have been harder to demonstrate benefits for these individuals had they been included. Others said they were too busy to take part, and this may have meant that they were already optimally active and hence unlikely to show benefits from an intervention aimed at increasing activity levels. It is important to target interventions of this kind appropriately.

### Feasibility and acceptability

It proved feasible to conduct the trial as intended. It was possible to recruit to target within the expected timescale, with two-thirds of those approached agreeing to participate, and attrition rates were very low. The partnership between the research team and ACGM was crucial and regular meetings ensured effective joint working. Embedding research within the centre, while sometimes initially met with caution, was in general viewed positively, and particularly so as a sense of familiarity developed. People attending the centre were willing to engage with the research, and those not directly participating nevertheless maintained an interest in the project. Participants were generally satisfied with the experience of participating, and although some of the cognitive, physical and health assessments were felt to be challenging, these were tolerated reasonably well. This supports the relevance of extending the model used here more widely in future work.

The goal-setting process also proved acceptable to participants, although some found identifying goals challenging. While the majority of goals were fully or partially achieved, a number were not achieved. It was noteworthy that although the domains in which goals were set were rated as important by participants, readiness to change was relatively low; in developing this approach further it may be necessary to explore ways of enhancing readiness to change and developing motivation as a preliminary stage in the goal-setting process. Nevertheless, ratings of performance, taken across all the goals set by each participant, did increase significantly. It is possible that including a specific requirement to set one goal in each of the two domains of physical and cognitive activity might have resulted in greater positive changes in these outcomes, but this had to be balanced against participant preference and choice, and participants were not restricted to setting goals in these areas. They had the option to choose goals in any of four domains, which also included the areas of physical health and diet, and social engagement. The qualitative investigation revealed that some participants allocated to the control condition felt that they already tended to set themselves goals and therefore would not have benefitted further from support with goal-setting. Similarly, there were diverse views about how welcome mentoring phone calls might be. This suggests that in practice offering a range of options may be necessary for optimal results.

A frequent concern with research in this domain relates to the ability to reach those groups of potential participants who are most in need or most likely to benefit [73,74]. The Agewell centre model aims to be socially inclusive, and centres are established in places where there is seen to be a need. In this case, the relative remoteness and isolation of the rural community and the relative lack of opportunities for activity engagement were key factors. It is a strength of the study that the majority of participants had no formal qualifications, had been in skilled or semi-skilled but not managerial or professional occupations, were judged to have poor social capital and moderate to high levels of material deprivation, and were considered disadvantaged in terms of frequency of contact with others.

One important limitation that emerged during the course of the trial was the difficulty we experienced in getting men to participate. There may be gender differences in response to intervention approaches aimed at increasing activity levels [75]. Those attending the centre were predominantly female, and this was reflected in the study sample. Men did attend certain activities, such as the computer classes, but tended to restrict their attendance to these rather than becoming involved more widely, and hence there were limited options for recruiting men into the study. Almost all the male participants attended the centre with their wives, and husbands and wives were randomised together to avoid cross-contamination between conditions, which given the small sample size resulted in a somewhat uneven distribution of males across groups. Based on our results, therefore, it is not possible to draw conclusions about the extent to which men may benefit from this kind of approach, since the majority of participants were female. In future work of this kind there will be a need to find ways to engage and involve male participants.

## Conclusions

The results demonstrate the feasibility of this relatively low-cost, theoretically-based goal-setting approach and the possibility of bringing about changes in behaviour and lifestyle which impact on key outcomes and are relevant to risk reduction. Future work will need to determine what changes can be reliably observed in large samples, whether and how such changes can be sustained over a longer period, and whether observed changes do indeed result in delayed onset or prevention of cognitive impairment and dementia. However, the findings from this study suggest that a brief, low-cost goal-setting intervention is feasible and acceptable, and has the potential to achieve increased activity engagement. Adding low-cost behaviour change approaches, or integrating these into newly-developing initiatives, could maximise the health-promoting benefits of existing community resources.
